# Reviews

**Published:** 1879-10

**Authors:** 


### Reviews.

**Clinical Medicine; a Systematic Treatise on the Diagnosis and Treatment of Diseases.** Designed for the use of students and practitioners of medicine. By AUSTIN FLINT, M. D. Philadelphia: Henry C Lea. 1879. 795 pp.

In the preface to this admirable work, Dr. Flint says: "The belief that a work, devoted to the diagnosis and treatment of diseases, would be of use to the medical student, in his clinical studies, and useful as a book of reference to the practitioner, has led to the preparation of this volume. The plan of the work and the arrangement of diseases have been made with special reference to clinical medicine."

With this object in view, the author classifies diseases into the Respiratory, Circulatory, Digestive, Urinary and Nervous. The space devoted to the neuroses, on account of the activity of pathological and clinical research, given to this province of medicine, is the largest. By limiting the discussion of each subject, the author has condensed within a moderate-sized volume a concise treatise on practical medicine.

Dr. Flint, more than any other medical writer on this side of the Atlantic, combines in all his works, not only the beauty and elegance of diction of the essayist, thus making the productions of his pen almost classical, but elucidates with singular clearness every point and feature of the subject under consideration. Not unlike all his writings, the present volume is a model of literary excellence, and adds to the deservedly high reputation of the accomplished author.

The general practitioner will find in this volume invaluable assistance, while his limited leisure will yield profitable results if devoted to the attentive perusal of its pages. L.

---

**Sexual Neuroses.** By J. T. KENT. St. Louis: Maynard & Fedford, Printers and Binders.

This is a small work of 144 pages octavo. The title gives an idea of the contents, this being that usually found in writings upon similar subjects. We find hardly anything absolutely new in its pages, and while it covers the ground fairly we can hardly consider that there exists so great a necessity for books of this nature as the writer seems to believe. V. P.

---

**Materia Medica and Therapeutics.** By CHARLES D. F. PHILLIPS, M. D., F. R. C. S. E. Edited and adapted to the United States Pharmacopœa. By H. G. PIFFARD, M. A., M. D. New York: William Wood & Co.

The work is limited to the consideration of drugs of vegetable origin, and while not pretending to original research as to abstract questions, it does contain a great deal of practical information as to the use of drugs, much of it founded, as the author states, on the results of his own observations. Some of his conclusions are open to a "reasonable doubt;" as, for instance, the statement that "Belladonna is of almost unexampled importance," that "with the exception of opium there is probably no vegetable medicine so important in existence." The book presents, however, an extensive array of facts respecting the action of drugs, and much valuable information not found in the ordinary text-books, and we consider it well worthy of a place in "Wood's Library of Standard Medical Authors," of which valuable series it forms the eighth volume. D.

---

**The American Journal of Otology.**

This journal comes to us filled with material fresh and practical, and it is not surprising when we consider that the editorial staff is composed of some half a dozen of the most eminent aural surgeons in America. In conjunction with these, Prof. Mayer, of Hoboken, furnishes articles relating to acoustics, which can not fail to interest even the general reader. Every one who has seen the admirable little volume on Sound, in the "Experimental Science Series for Beginners," must have been impressed by the rare faculty which this man possesses of simplifying apparently difficult subjects. And this effort to popularize special branches of science is one of the sure indications of present progress. Not many years ago it was a current belief among the younger students at the Harvard Medical School that no one knew anything about the ear except God and Dr. Blake, and no one seemed to care about it either.

To-day we have two earnest workers in Boston, with others in different parts of the country, presenting the various phases of otology in a manner to interest a large circle of readers. Some of the articles are so thoroughly technical as to be of interest only to the specialist, giving results of deep researches, in departments of the subject hitherto unexplored. But the object of the journal is evidently not so much to record original observation as it is to furnish the profession at large with a practical view of the most recent discoveries in otology. H.

---

**A Text Book of Physiology.** By J. FULTON, M. D., M. R. C. S. Lindsay & Blakiston.

The one who buys this work is pretty sure of his money's worth of the essence of physiology. The author does not go into any tiresome details of the subject, but presents its main outlines in a concise and forcible manner. The microscopic structure of the various organs and tissues is described at length, thus giving the student a clearer idea of the manner in which their functions are performed, and if the numerous wood-cuts were more carefully executed, it might serve as a manual of histology as well. The practical phase of every question is, however, fully shown. The opportunity is not lost to explain the various tests for sugar and albumen, the uses of the sphygmograph, spirometer, and other methods or appliances which are of direct importance to the physician. The writer attempted to present "A well-digested text book on this subject, adapted to the wants of the advanced medical student and the general practitioner," and he has succeeded. H.

**Guide to the Examination of the Urine, with special reference to Diseases of the Urinary Apparatus.** By K. B. HOFFMANN, Professor at the University of Graz, and R. ULTZMAN, University of Vienna. From the Second Edition. Translated by R. FORCHEIMER, M. D., Professor of Medicine and Chemistry, Medical College of Ohio. Cincinnati: Peter G. Thompson. Cloth, $1.50; Leather, $2.00.

We most cordially recommend this little book to medical men. While, as the authors state, "It is not intended for the physiological chemist, or for those who make a special study of animal chemistry," we consider it one of the best books on urinalysis for the practical physician, who desires to determine the physical character and chemical constituents of the urine, *so far as they are important to him.* The numerous illustrations which accompany the text are well drawn and valuable, though rather roughly executed. The book is well printed, and its appearance is creditable to the publishers. D.

---

**Statistics of Placenta Prævia, collected from the Practice of Physicians of the State of Indiana.** By ENOCH W. KING, M. D., Galena, Ind.

A pamphlet with this title has been received, with the request that it be reviewed. We have looked its pages over carefully, and are prompted to speak well of it. The doctor was led to undertake the labor of collection and classification through an expression made by Dr. Sutton, in a paper read before the State Society, in which he says, "That many of our physicians, whose range of practice is principally confined to the country, have a large amount of valuable unpublished experience on this subject, which if collected in the form of statistics would throw light on the frequency of the occurrence of placenta prævia, the modes of treatment principally adopted, and the fatality attending this accident within our State."

The one hundred and twelve cases have been thoroughly analyzed under about twenty heads. The article adds valuable information to what is already known upon this subject, and we throw out the suggestion that here is a good field for others to follow the example of Dr. King, and that not alone as regards the subject of placenta prævia. V. P.

---

**Manual of Midwifery for Midwives and Medical Students.** By FANCOURT BARNES, M. D., M. R. C. P., &c. With illustrations. Philadelphia: Henry C. Lea.

A very neat and really valuable little book of about 200 pages octavo. It is, as the title indicates, simply an introductory work, but is one entitled to great praise. It compares very favorably with a similar work, viz.: Swain's Obstetric Aphorisms. Its numerous illustrations are in many instances copied from the work of his father, Dr. Robert Barnes, on Obstetric Operations. We recommend it to those for whom it is more particularly designed, *i. e.*, the medical student. V. P.

